# Transcriptome Profiling of Tomato Uncovers an Involvement of Cytochrome P450s and Peroxidases in Stigma Color Formation

**DOI:** 10.3389/fpls.2017.00897

**Published:** 2017-05-31

**Authors:** Yan Zhang, Guiye Zhao, Yushun Li, Jie Zhang, Meijing Shi, Tayeb Muhammad, Yan Liang

**Affiliations:** ^1^College of Horticulture, Northwest A&F UniversityYangling, China; ^2^State Key Laboratory of Crop Stress Biology in Arid Region, Northwest A&F UniversityYangling, China

**Keywords:** cytochrome P450s, peroxidases, tomato, transcriptome, yellow stigma

## Abstract

Stigma is a crucial structure of female reproductive organ in plants. Stigma color is usually regarded as an important trait in variety identification in some species, but the molecular mechanism of stigma color formation remains elusive. Here, we characterized a tomato mutant, *yellow stigma* (*ys*), that shows yellow rather than typical green color in the stigma. Analysis of pigment contents revealed that the level of flavonoid naringenin chalcone was increased in the *ys* stigma, possibly as a result of higher accumulation of *p*-coumaric acid, suggesting that naringenin chalcone might play a vital role in yellow color control in tomato stigma. To understand the genes and gene networks that regulate tomato stigma color, RNA-sequencing (RNA-Seq) analyses were performed to compare the transcriptomes of stigmas between *ys* mutant and wild-type (WT). We obtained 507 differentially expressed genes, in which, 84 and 423 genes were significantly up-regulated and down-regulated in the *ys* mutant, respectively. Two cytochrome P450 genes, *SlC3H1* and *SlC3H2* which encode *p*-coumarate 3-hydroxylases, and six peroxidase genes were identified to be dramatically inhibited in the yellow stigma. Further bioinformatic and biochemical analyses implied that the repression of the two *SlC3Hs* and six *PODs* may indirectly lead to higher naringenin chalcone level through inhibiting lignin biosynthesis, thereby contributing to yellow coloration in tomato stigma. Thus, our data suggest that two *SlC3Hs* and six *PODs* are involved in yellow stigma formation. This study provides valuable information for dissecting the molecular mechanism of stigma color control in tomato.

**Statement**: This study reveals that two cytochrome P450s (SlC3H1 and SlC3H2) and six peroxidases potentially regulate the yellow stigma formation by indirectly enhancing biosynthesis of yellow-colored naringenin chalcone in the stigma of tomato.

## Introduction

Pollination is a crucial event in sexual reproduction that sustains lifecycle of flowering plants on the face of the earth. The stigma, which functions as the recipient of pollen, shows wide variation in colors similar to flowers. Different colors of flowers and those of stigmas are attributed to accumulation of diverse pigments, which may attract specific pollinators to facilitate pollination. For example, in some species, such as *Arabidopsis* and tomato (*Solanum lycopersicum*), the color of stigma is green, whereas that in cucumber (*Cucumis sativus* L.) is yellow. Furthermore, the stigma color is often regarded as an important trait in variety identification in some crop plants such as rice (*Oryza sativa*). For instance, in many rice cultivars of Asia, the stigmas are colorless, but in most of wild germplasms, those are colored, signifying their involvement in the process of domestication (Han et al., 2006). So far, extensive studies have confirmed that the flower colors are due mainly to flavonoids, carotenoids and betalains, in which, the flavonoids are the most crucial (Grotewold, 2006; Tanaka and Brugliera, 2013; Gao et al., 2016). However, little is known about the involvement of pigments, especially flavonoids, in the formation of color in the stigma.

Flavonoids, a large group of plant polyphenols, are widely distributed throughout the plant kingdom. Until now, over 10,000 structures of flavonoids have been identified in plants (Julkunen-Tiitto et al., 2015). This diversity arises from combinatorial modifications of the basic flavonoid scaffold by decorating enzymes such as glycosyl, acyl, and methyl transferases, and also due to structural differences in the basic flavonoid skeleton such as isoflavonoids (Mehrtens et al., 2005). In addition to their functions as floral pigments, flavonoids play important roles in many other aspects of plant growth, development and responses to environmental stimuli, such as regulation of auxin transport, male fertility, pollination, pathogen resistance, and UV-light protection (Harborne and Williams, 2000; Zhang et al., 2015). Furthermore, flavonoids are good antioxidants and have been demonstrated to promote human health and reduce the primary risk factor for some diseases when consumed as foods from plant origin (Hannum, 2004; Hooper and Cassidy, 2006; Tapas et al., 2008; Martin et al., 2011; Li et al., 2015). Flavonoids can be classified into different subgroups: chalcones, flavones, flavonols, flavanones, isoflavones, flavanols, and anthocyanins, in which, the chalcones function as yellow pigments in many flowers, the anthocyanins usually display red, purple, and blue colors, while several other classes such as flavonols and flavanols serve as co-pigments (Tanaka et al., 2008; Petrussa et al., 2013).

**FIGURE 1:**
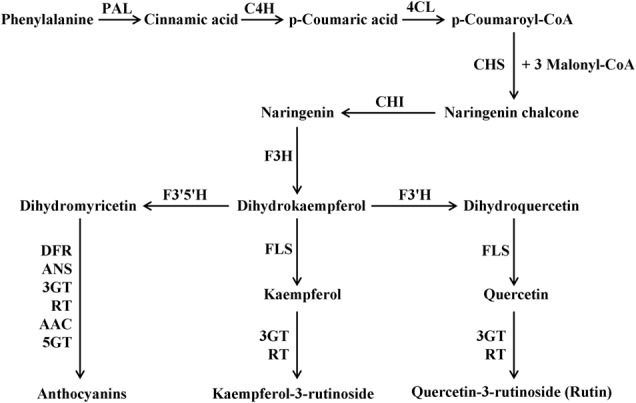
Simplified overview of the flavonoid biosynthetic pathway in tomato. PAL, phenylalanine ammonia-lyase; C4H, cinnamate 4-hydroxylase; 4CL, 4-coumarate:CoA ligase; CHS, chalcone synthase; CHI, chalcone isomerase; F3H, flavanone-3-hydroxylase; F3′H, flavonoid 3′-hydroxylase; F3′5′H, flavonoid 3′,5′-hydroxylase; FLS, flavonol synthase; DFR, dihydroflavonol reductase; ANS, anthocyanidin synthase; 3GT, flavonoid-3-*O*-glucosyltransferase; RT, flavonoid 3-*O*-glucoside-rhamnosyltransferase; AAC, anthocyanin acyltransferase; 5GT, flavonoid-5-glucosyltransferase.

The flavonoid biosynthetic pathway has been well established (**Figure 1**), and it is conserved among seed plants (Tanaka et al., 2008; Buer et al., 2010; Petrussa et al., 2013; Saito et al., 2013; Weston and Mathesius, 2013). Flavonoids are biosynthesized along the general phenylpropanoid pathway in the cytosol (Winkel, 2004). Previous studies have shown that the enzymes involved in the biosynthetic pathway form a multienzyme complex (metabolon) via protein–protein interaction and bind to the ER membrane (Grotewold, 2006; Ono et al., 2006; Kuhn et al., 2011). These enzymes belong to several enzyme families, such as cytochrome-P450, OGD, and GT (Tanaka, 2006; Tanaka et al., 2008). The genes encoding these biosynthetic enzymes of the pathway, such as *CHS, CHI, F3′H, F3′5′H, FLS, DFR, ANS, 3GT*, flavonoid 3-*O*-glucoside-rhamnosyltransferase (*RT*) and *5GT* have been cloned and analyzed in different plant species (Shirley et al., 1995; Pourcel et al., 2005; Buer et al., 2007; Owens et al., 2008a,b; Buer and Djordjevic, 2009; Falcone Ferreyra et al., 2015). Besides these biosynthetic genes, combinations of the R2R3-MYB, bHLH and WDR factors and their interactions are also involved in regulation of flavonoid biosynthesis at the transcriptional level. This model has been well understood in *Arabidopsis*, tomato, maize (*Zea mays*) petunia (*Petunia hybrida*) and some other plants (Koes et al., 2005; Ramsay and Glover, 2005; Morita et al., 2006; Gonzali et al., 2009; Xu et al., 2015). MYB and bHLH transcription factors (TFs) are the largest families among plant TFs and are found in all eukaryotes (Heim et al., 2003; Dubos et al., 2010; Feller et al., 2011), whereas WDR proteins are pleiotropic, which participate in multiple processes such as flavonoid biosynthesis, the fate of multiple epidermal cells and the formation of trichomes and root hairs (Tanaka et al., 2008; Li et al., 2014). Interestingly, these three proteins can form a ternary complex named as MBW (MYB-bHLH-WDR) (Baudry et al., 2004). It is believed that the MBW complex is directly responsible for the activation of flavonoid LBGs expression (Gonzalez et al., 2008; Thevenin et al., 2012; Xu et al., 2014, 2015).

Tomato has been widely cultivated as an important vegetable crop around the world. Although the flavonoid biosynthetic pathway is well established in tomato (Ye et al., 2015), the majority of the studies are focused on flavonoids-regulated color formation in tomato fruits. Until now, around 70 flavonoids have been identified in tomato fruit, which are predominantly accumulated in the fruit peel (Bovy et al., 2002; Colliver et al., 2002; Iijima et al., 2008; Ballester et al., 2010). The major flavonoids in tomato fruit are naringenin chalcone, quercetin, kaempferol, and their conjugated forms such as different glycosides (Moco et al., 2006). Among them, yellow-colored naringenin chalcone, one of the most abundant flavonoids in tomato fruit, is accumulated in the cuticle during the fruit ripening and thus determines the yellow color in the peel at the breaker stage. In addition, quercetin-3-rutinoside (rutin) and kaempferol-3-rutinoside are also distributed in the peel of ripen fruit (Ballester et al., 2010).

Despite the importance of the stigma as an essential structure of female reproductive organ, little is known about the mechanism of the regulation of stigma color in plants. Moreover, to the best of our knowledge, there are no identified tomato mutants with different colors of stigmas. In this study, through EMS mutagenesis, we identified a mutant with unusual yellow stigma color. This mutant was named as *ys*. We investigated the pigment contents in the stigmas of *ys* mutant and WT (green stigma), and performed comparative transcriptome profiling analyses to elucidate genes and gene networks involved in yellow stigma formation in tomato. Our study revealed that the accumulation of yellow-colored flavonoid naringenin chalcone was significantly increased in the yellow stigma, and two cytochrome P450 genes, *SlC3H1* and *SlC3H2* that encode the *p*-coumarate 3-hydroxylases, and six POD genes might participate in yellow stigma formation through indirect regulation of the naringenin chalcone level in the *ys* mutant. These results provide valuable information for dissecting the molecular mechanism of yellow stigma formation in tomato.

## Materials and Methods

### Plant Materials and Growth Conditions

The *ys* mutant of tomato was generated in the background of the inbred line TTD302A through EMS mutagenesis using Saito’s method (Saito et al., 2011), and stabilized via six generations of selfing prior to this study. The seeds of *ys* mutant and WT were germinated on wet filter paper in a Petri dish at 28°C in dark for 3 days. Then the resulting seedlings were grown in a growth chamber under a 16 h/8 h (light/dark) photoperiod with 25/16°C temperatures, respectively. Upon four true-leaf stage, plants were transferred to a greenhouse in the experimental field of the Northwest A&F University. Pest control and water management were carried out according to standard practices.

### Measurement of Chlorophyll, Carotenoid, and Flavonoid Levels

The fresh stigmas at the anthesis stage were collected from WT and *ys* mutant at the same time on the same day, and then immediately used to pigment measurement. Total chlorophylls and carotenoids were extracted and quantified by spectrophotometric method as described previously (Lichtenthaler, 1987; Bou-Torrent et al., 2015).

Flavonoids and other polyphenols extraction and analyses were carried out according to Bino et al. (2005) with some modifications. The 100 mg frozen tomato stigmas powder was extracted with 80% methanol for 12 h at 4°C followed by 30 min sonication. The mixtures were then centrifuged at 12,000 rpm for 10 min at 4°C and the supernatants were filtered (0.2 μm). The extraction was repeated once by the above method. The samples were analyzed and identified using a Waters Alliance 2695 HT HPLC system coupled with a Q-TOF Mass Spectrometer (Waters-Micromass, Milford, MA, United States). The 20 μL of sample extract was injected and separated using a Luna C18 reverse-phase analytical column (3 μm, 150 mm × 2.1 mm; Phenomenex) at 40°C and a gradient of 5–50% acetonitrile in 0.1% formic acid at a flow rate of 0.3 mL min^-1^. The eluted compounds were detected at 200–600 nm using a 996 PDA detector (Waters, Milford, MA, United States), followed by the mass spectrometer equipped with an electrospray ionization (ESI) source. The conditions for LC-MS runs were as follows: desolvation temperature was 250°C with a nitrogen gas flow rate of 10 L min^-1^ and capillary voltage was 3 kV; source temperature was 120°C; cone voltage was 35 eV with 1 L min^-1^ gas flow and collision energy was 5 eV in positive ion mode or 10 eV in ion mode. Ions in the *m/z* range 100–1500 were detected using a scan time of 0.9 s and an interscan delay of 0.1 s. The flavonoid compounds and other polyphenols were identified using retention times obtained by authentic standards and mass calculated for the ion (*M* + *H*)^+^ (Supplementary Table S1), and quantified by comparing the area of each individual peak with the standard curves obtained from the pure compounds. All measurements were repeated with three independent biological samples, and each sample was assayed in triplicate.

### Lignin Content Determination

The stigmas at the anthesis stage from WT and *ys* mutant were harvested and freeze-dried in liquid nitrogen. The lignin content was determined using the acetyl bromide method as described previously with some modifications (Yan et al., 2012). The samples were ground into powder, then 10 mg of powder was rinsed four times with 95% ethanol and twice with the mixture of 100% ethanol and *n*-hexane (1:2 in volume). The precipitate was collected, dried at 60°C and then suspended in 2 mL of 25% acetyl bromide in glacial acetic acid. After incubation at 70°C for 30 min, 0.9 mL 2 M NaOH was added, followed by 2 mL glacial acetic acid and 0.1 mL 7.5 M hydroxylamine hydrochloride. The mixture was centrifuged at 4,500 rpm for 5 min and the supernatant was collected, then the absorbance was measured at 280 nm. The lignin content was expressed as Absorption 280 on a fresh weight basis. All measurements were performed with three biological samples, and each sample was assayed in triplicate.

### Differentially Gene Expression (DGE) Library Construction and Sequencing

Young stigmas of about 1 mm in length were collected from WT and ys mutant at the same time on the same day. Samples were immediately frozen in liquid nitrogen and stored at -80°C for RNA-Seq analyses. Total RNA was isolated using the RNA extraction kit (Promega, United States). RNA quality was checked by RNase-free agarose gel electrophoresis to avoid possible degradation and contamination, and then verified using Agilent 2100 Bio-analyzer (Agilent Technologies, Santa Clara, CA, United States). Next, Poly (A) mRNA was isolated using oligo-dT beads (Qiagen, Germany), and then broken into short fragments by adding fragmentation buffer. First-strand cDNA was synthesized using random hexamer-primed reverse transcription, followed by the synthesis of the second-strand cDNA using RNase H and DNA polymerase I. The cDNA fragments were purified using a QIA quick PCR extraction kit, and then washed with EB buffer for end reparation poly (A) addition and ligated to sequencing adapters. Following agarose gel electrophoresis and extraction of cDNA from gels, the cDNA fragments were purified and enriched by PCR to construct the final cDNA library, which was then sequenced on the Illumina sequencing platform (Illumina HiSeq^TM^ 2500) using the paired-end technology. Three biological replicates were performed for each line from WT and ys mutant, thus six DGE libraries were generated and sequenced.

### Bioinformatics Analysis of DGE Data

Raw reads were filtered to remove low quality sequences (there were more than 50% bases with quality lower than 20 in one sequence), reads with more than 5% N bases (bases unknown) and reads containing adaptor sequences through the Perl program. Then the clean reads were mapped to the tomato reference genome using TopHat2 (Consortium, 2012; Kim D. et al., 2013), allowing up to one mismatch. Unigenes mapped by at least one read, in at least one sample, were identified for further analysis. The DEGs were identified using the R package edgeR (Robinson et al., 2010). The expression level of each unigene was calculated and normalized to FPKM. The FDR was used to determine the threshold of the P-value in multiple tests. In our study, the FDR < 0.05 and fold change > 2 were used as significance cut-offs of the gene expression differences.

Sequencing data were deposited to the Short Read Archive (SRA) database at the National Center for Biotechnology Information (NCBI) under the accession number SRP080654.

Further, the DEGs were used for GO and KEGG enrichment analyses according to Zhang et al. (2013). GO terms with corrected P-value < 0.05 and KEGG pathways with P-value < 0.05 were considered significantly enriched by differential expressed genes.

### Quantitative Real-Time PCR (qRT-PCR) Verification

Quantitative real-time PCR assays were performed using the independent tomato stigmas in the same developmental stage as those used for DGE analysis. Total RNA was isolated using the RNA extraction kit (Promega, United States), and cDNA was synthesized using MultiScribe reverse transcriptase (Applied Biosystems, United States). qRT-PCR was carried out using SYBR^®^ *Premix Ex Taq^TM^* from TaKaRa (China) on an Applied Biosystems 7500 real-time PCR system (Applied Biosystems, United States). The tomato *EF-1α* gene was used as reference gene to normalize the expression data. Each qRT-PCR experiment was repeated with three biological samples, and each sample was assayed in triplicate. The relative expression of genes was calculated using the 2^-ΔΔ^*^C^*^t^ method and standard error was calculated between three biological replicates. The gene specific primers for qRT-PCR are listed in Supplementary Table S2.

## Results

### Specific Flavonoid Accumulation is Responsible for Yellow Stigma Phenotype in the *ys* Mutant

**FIGURE 2:**
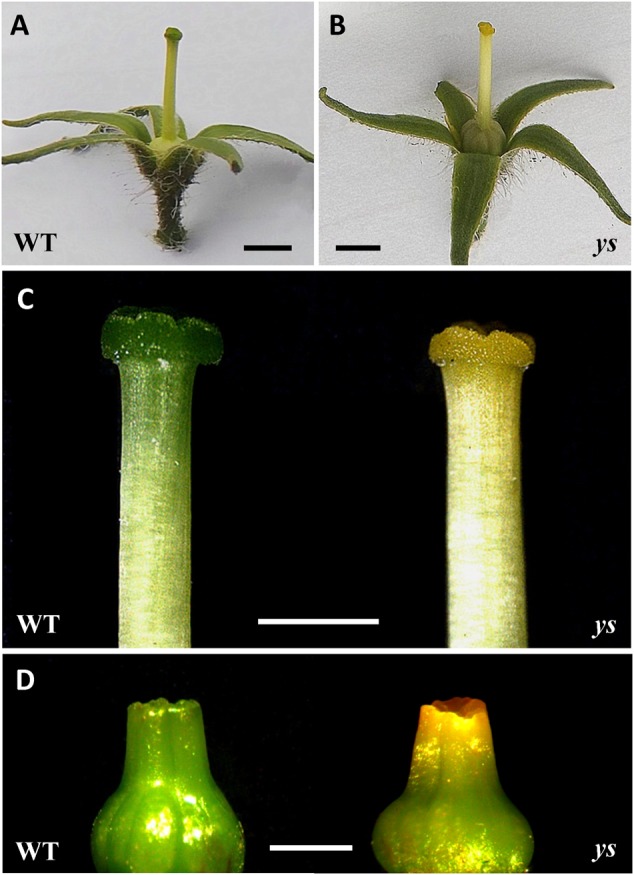
Morphological characterization of yellow stigma phenotype in the *ys* mutant. **(A,B)** Phenotype of flowers after removal of petals and stamens in WT **(A)** and *ys* mutant **(B)**. **(C)** Stigmas of WT or *ys* mutant flowers at the anthesis stage. **(D)** Young stigmas (length around 1 mm) in WT or *ys* mutant. Bars = 2 mm **(A,B)** and 1 mm **(C,D)**.

While the WT tomato plant was characterized by typical green stigma, the *ys* mutant, generated through EMS mutagenesis, showed unusual “yellow stigma” phenotype (**Figures 2A–C**). Notably, this stigma color difference between WT and *ys* mutant was apparent even in the very early stage of stigma development (stigma length around 1 mm) (**Figure 2D**).

**FIGURE 3:**
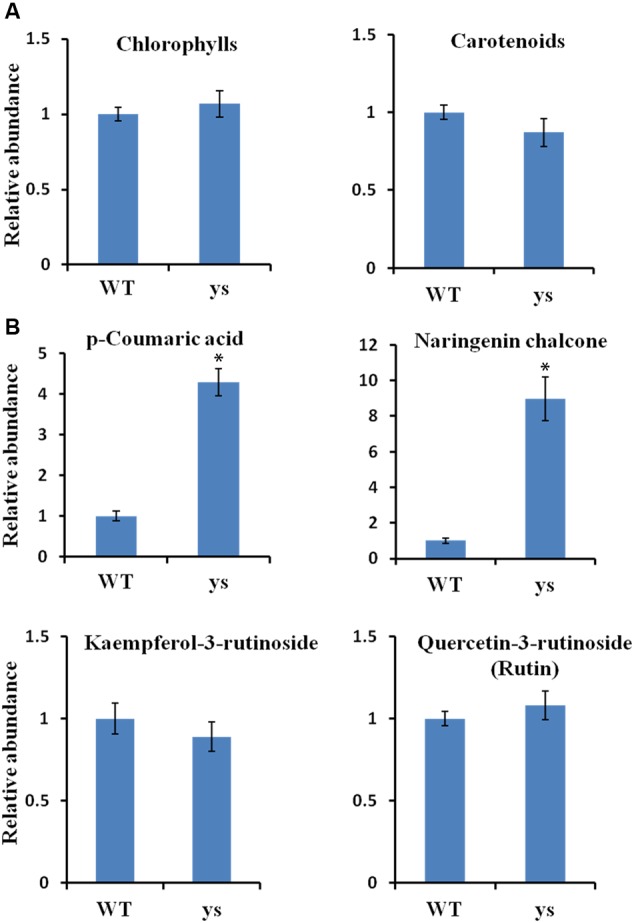
Pigments accumulation in stigmas at the anthesis stage of WT and *ys* mutant. **(A)** Total chlorophyll and carotenoid levels in stigmas of WT and *ys* mutant. **(B)** Flavonoid levels in stigmas of WT and *ys* mutant. Values are means ± SE from three biological replicates (*n* = 3). Asterisks indicate the significant differences (*P* < 0.01) between WT and *ys* mutant determined by Duncan’s test.

To explore important pigments responsible for the “yellow stigma” phenotype, we quantified the contents of chlorophylls, carotenoids and flavonoids in the stigmas of WT and *ys* mutant during anthesis stage. To our surprise, no significant changes were noticed in both total chlorophyll and carotenoid levels between WT and *ys* mutant (**Figure 3A** and Supplementary Table S3). However, remarkable differences were found for some polyphenol contents in the flavonoid biosynthetic pathway. For instance, the yellow stigma accumulated 4.3- and 8.9-fold increased *p*-coumaric acid and naringenin chalcone, respectively. However, the levels of kaempferol-3-rutinoside and quercetin-3-rutinoside (rutin) in stigmas were not changed between WT and *ys* mutant (**Figure 3B** and Supplementary Table S3). It is worth mentioning that the *p*-coumaric acid is a biosynthetic precursor of naringenin chalcone (**Figure 1**) (Petrussa et al., 2013; Saito et al., 2013; Falcone Ferreyra et al., 2015). Therefore, it is highly plausible that the higher accumulation of *p*-coumaric acid in the *ys* mutant led to an increased naringenin chalcone level. These results suggested that increased accumulation of yellow-colored flavonoid naringenin chalcone may be responsible for the yellow stigma phenotype in the *ys* mutant.

### Identification of Differentially Expressed Genes (DEGs) of Stigmas from WT and *ys* Mutant

To identify genes and gene networks that are involved in the formation of yellow stigma in tomato, we performed genome-wide expression analyses to compare the transcriptome profiles of the stigmas between WT and *ys* mutant through the DGE approach (Eveland et al., 2010). Given that the phenotypic changes occurred in the early stage (**Figure 2D**), we chose young stigmas (length around 1 mm) for RNA-Seq analyses. In this project, 42.3–50.8 million raw reads from each DGE library were generated. After removal of adapter sequences and low-quality reads, we obtained 41.8–50.1 million high-quality clean reads with a total of 5.2–6.3 billion nucleotides. Among these clean reads, the percentage of Q20 (base quality more than 20) and GC was 94.4–94.8% and 42.8–43.0%, respectively (Supplementary Figure S1 and Table S4). Further, we clustered the clean reads into unique reads, which were mapped to the tomato genome using TopHat2 (Consortium, 2012; Kim D. et al., 2013). In the six DGE libraries, about 91.2–92.9% of clean reads from RNA-Seq data were mapped uniquely to the tomato genome (Supplementary Table S4).

Based on deep sequencing, 23,978 genes were detected in all libraries (Supplementary Table S5). We used the R package edgeR to identify the DEGs (Robinson et al., 2010). The expression level of each gene was normalized to FPKM. Using FDR < 0.05 and fold change > 2 as the significance cut-offs, we obtained 507 DEGs, in which 84 genes were significantly up-regulated and 423 genes were dramatically down-regulated in the stigmas of *ys* mutant as compared to those of WT plants (Supplementary Table S6). To validate the RNA-Seq data, we performed qRT-PCR assays using the independent tomato stigmas in the same developmental stage as those used for DGE analysis. Sixteen DEGs, including 6 up-regulated genes and 10 down-regulated genes, were randomly chosen for qRT-PCR analysis. As shown in **Table 1**, the qRT-PCR data were in full agreement with the RNA-Seq data in terms of relative fold change in the expression of these 16 genes between WT and *ys* mutant (Pearson correlation coefficient 0.969, *P* = 6.3E - 10), suggesting that the RNA-Seq results were highly reliable.

**Table 1:** qRT-PCR verification of differentially expressed genes identified by RNA-Seq.

Gene ID	Gene annotation	DGE FDR	DGE fold change (*ys* vs. WT)	qRT-PCR fold change (*ys* vs. WT)
Solyc12g008900	CKX6 (cytokinin oxidase 6)	1.02E - 04	21.32	14.84 @ 0.72
Solyc03g093890	MYB52 (R2R3-MYB domain protein 52)	3.65E - 02	14.50	11.08 @ 0.82
Solyc08g076820	bHLH146 (bHLH family protein 146)	4.60E - 02	13.29	18.56 @ 1.24
Solyc08g074620	PPO (polyphenol oxidase)	7.74E - 07	10.62	7.75 @ 0.67
Solyc10g055760	NAC6 (NAC domain protein 6)	6.36E - 06	10.27	6.88 @ 0.84
Solyc06g064840	AG (agamous MADS-box protein)	7.67E - 04	10.13	11.27 @ 1.50
Solyc10g078220	CYP450 (cytochrome P450)	5.23E - 07	–139.82	–62.69 @ 5.34
Solyc10g078230	CYP450 (cytochrome P450)	4.24E - 04	–130.78	–60.26 @ 6.35
Solyc07g056510	GST (glutathione *S*-transferase)	9.84E - 03	–86.87	–39.51 @ 4.43
Solyc07g017880	POD (peroxidase)	1.50E - 08	–54.91	–43.28 @ 3.39
Solyc02g084790	POD (peroxidase)	3.69E - 02	–28.24	–19.72 @ 4.25
Solyc01g109120	WD40 (WD40 repeat protein)	7.47E - 03	–8.83	–9.56 @ 1.58
Solyc07g056670	GA2OX2 (gibberellin 2-oxidase 2)	6.86E - 07	–8.14	–3.97 @ 0.56
Solyc06g083170	bHLH049 (bHLH family protein 049)	2.80E - 05	–4.96	–4.69 @ 0.58
Solyc01g100460	bZIP (bZIP domain protein)	5.00E - 03	–3.38	–5.26 @ 0.79
Solyc04g077010	RLK (receptor like kinase)	1.89E - 02	–2.98	–2.36 @ 0.27

*The relative transcript level of each gene was expressed as the fold change between WT and *ys* mutant in the RNA-Seq data and qRT-PCR analyses. The tomato *EF-1α* gene was used as reference gene. The values shown represent the means ± SE from three biological replicates (*n* = 3).*

### Cytochrome P450s and Peroxidases Are Involved in the Formation of Yellow Stigma in Tomato

**FIGURE 4:**
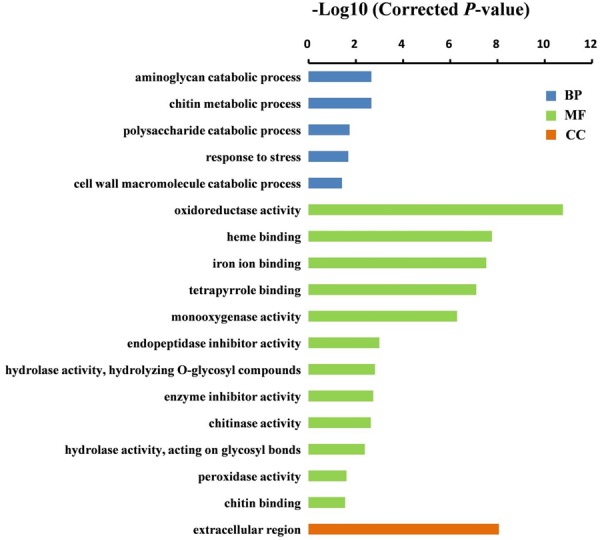
GO terms that were significantly enriched in the DEGs between WT and *ys* mutant stigma. The results were summarized in three main categories: biological process (BP, blue), molecular function (MF, green), and cellular component (CC, orange). GO terms were sorted based on corrected *P*-value, and the corrected *P*-value < 0.05 was used as the significance cut-off.

In order to understand the expression profiles and potential functions of DEGs identified by DGE, GO term enrichment analysis (corrected *P*-value < 0.05) was performed. The DEGs were classified into cellular component, biological process and molecular function categories (Supplementary Table S7). As shown in **Figure 4**, there was only one group “extracellular region” (*P* = 8.8E - 09) in the cellular component category. The GO terms of catabolic and metabolic processes were dominantly enriched in the biological process group. In the molecular function category, the top three significantly enriched GO terms were “oxidoreductase activity” (*P* = 1.7E - 11), “heme binding” (*P* = 1.7E - 08) and “iron ion binding” (*P* = 3.0E - 08). Interestingly, these three groups all shared 23 genes encoding cytochrome P450s (CYP450), a large family of enzymes that play a crucial role in the biosynthesis of flavonoids that are responsible for flower colors (Tanaka, 2006; Tanaka and Brugliera, 2013), and 10 genes encoding PODs (Supplementary Table S7), however, most of those genes were down-regulated in the stigmas of *ys* mutant compared to WT (**Table 2**). For example, the expression levels of two *CYP450* genes, *Solyc10g078220* and *Solyc10g078230*, and two *POD* genes, *Solyc07g017880* and *Solyc02g084790*, decreased 139.8-, 130.8-, 54.9- and 28.2-fold, respectively, in the *ys* mutant compared with those in the WT, and qRT-PCR verification revealed the same expression pattern (**Table 1**). These data indicated that the cytochrome P450s and PODs may play important role in the formation of yellow stigma in tomato.

**Table 2:** List of differentially expressed cytochrome P450 and POD genes identified by DGE in the stigmas of *ys* mutant and WT.

Gene ID	Fold Change (ys/WT)	FDR
**Cytochrome P450s**		
Solyc03g122350	16.42	3.41E - 09
Solyc03g114940	14.32	7.69E - 06
Solyc12g006860	8.57	5.25E - 03
Solyc10g078220	–139.82	5.23E - 07
Solyc10g078230	–130.78	4.24E - 04
Solyc04g083140	–128.18	9.91E - 10
Solyc07g055560	–65.55	6.09E - 19
Solyc12g045020	–48.07	1.78E - 02
Solyc06g066230	–46.11	4.22E - 04
Solyc08g075320	–45.88	4.24E - 08
Solyc08g079420	–27.85	1.44E - 05
Solyc07g052370	–23.20	3.81E - 03
Solyc06g076160	–20.46	1.39E - 03
Solyc04g079660	–19.01	4.01E - 04
Solyc07g062500	–14.75	5.62E - 03
Solyc04g050620	–13.33	6.07E - 09
Solyc10g083690	–12.94	2.94E - 07
Solyc03g112030	–10.56	4.58E - 03
Solyc04g079640	–4.93	1.37E - 04
Solyc07g055460	–4.52	9.34E - 03
Solyc04g078900	–2.78	1.89E - 02
Solyc09g098610	–2.75	1.27E - 02
Solyc10g087040	–2.25	2.34E - 02
**Peroxidases**		
Solyc01g006300	–9990.00	4.49E - 03
Solyc02g087070	–102.67	3.03E - 03
Solyc07g017880	–54.91	1.50E - 08
Solyc02g094180	–53.14	5.27E - 06
Solyc01g105070	–43.48	1.76E - 06
Solyc02g084790	–28.24	3.69E - 02
Solyc10g076240	–21.33	2.98E - 07
Solyc03g006700	–9.45	5.00E - 03
Solyc02g079500	–3.04	1.77E - 02
Solyc04g071890	–2.17	4.26E - 02

### Two Cytochrome P450 Genes and Six Peroxidase Genes May Function As Negative Regulators in Yellow Stigma Formation in Tomato

**FIGURE 5:**
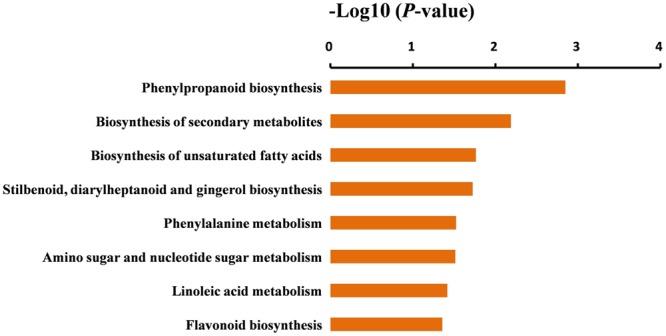
Significantly enriched KEGG pathways in the DEGs. KEGG pathways were sorted based on *P*-value, and the *P*-value < 0.05 was used as the significance cut-off.

**FIGURE 6:**
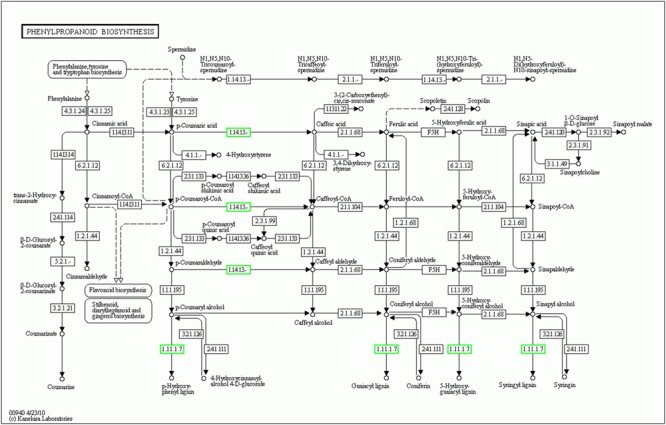
KEGG graph of phenylpropanoid biosynthesis pathway. Down-regulated and unchanged genes are shown in green and black boxes, respectively. “1.14.13.–” in the green boxes indicate the two cytochrome P450 genes *SlC3H1* (*Solyc10g078220*) and *SlC3H2* (*Solyc10g078230*). “1.11.1.7” in the green boxes represent the six POD genes (*Solyc07g017880, Solyc02g084790, Solyc10g076240, Solyc03g006700, Solyc02g079500, Solyc04g071890*).

To further identify the biological pathways that are responsible for the formation of yellow stigma in tomato, we mapped the detected DEGs to reference canonical pathways in the KEGG (Kanehisa et al., 2004), and then compared these with the whole transcriptome background to search for genes involved in the metabolic or signal transduction pathways that were significantly enriched. Using the *P*-value < 0.05 as the significance cut-off, 29 DEGs, including 3 up-regulated and 26 down-regulated genes, were assigned to 8 KEGG pathways (Supplementary Table S8). Among them, the “Phenylpropanoid biosynthesis” (*P* = 1.4E - 03), “Biosynthesis of secondary metabolites” (*P* = 6.5E - 03) and “Biosynthesis of unsaturated fatty acids” (*P* = 1.7E - 02) were the top three significantly enriched KEGG pathways (**Figure 5**). However, the “Flavonoid biosynthesis” pathway (*P* = 4.4E - 02), which was putatively associated with the yellow stigma phenotype in the *ys* mutant (**Figure 3B**), was also enriched, despite the *P*-value showed higher. Notably, two *CYP450* genes (*Solyc10g078220* and *Solyc10g078230*) and six *POD* genes (*Solyc07g017880, Solyc02g084790, Solyc10g076240, Solyc03g006700, Solyc02g079500*, and *Solyc04g071890*), whose expressions were dramatically down-regulated in the stigma of *ys* mutant (**Table 2**), were mapped into the “Phenylpropanoid biosynthesis” and “Biosynthesis of secondary metabolites” (Supplementary Table S8). Moreover, the two *CYP450* genes were also involved in the “Flavonoid biosynthesis” pathway (Supplementary Table S8). In these pathways, the two *CYP450* genes encoded the C3H which catalyze the *p*-coumaric acid, *p*-coumaroyl-CoA and *p*-coumaraldehyde to caffeic acid, caffeoyl-CoA and caffeyl aldehyde, respectively (**Figure 6** and Supplementary Figure S2) (Xue et al., 2014; Fornaléa et al., 2015), so, we named these two genes as *SlC3H1* (*Solyc10g078220*) and *SlC3H2* (*Solyc10g078230*). In addition, the six *POD* genes were involved in the last step of biosynthesis of lignin, an insoluble cell wall-associated polymer (**Figure 6**) (Quiroga et al., 2000; Higuchi, 2006).

**FIGURE 7:**
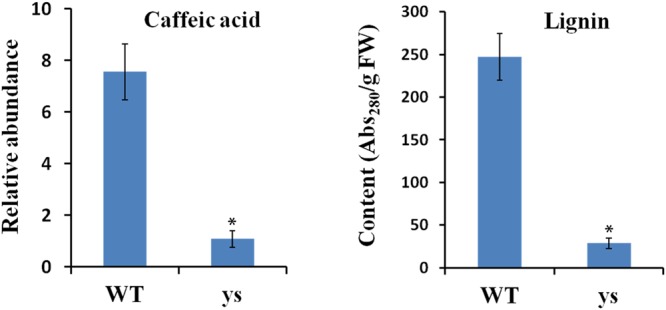
Caffeic acid and lignin contents in stigmas at the anthesis stage from WT and *ys* mutant. Values represent the means ± SE from three biological replicates (*n* = 3). Asterisks indicate the significant differences (*P* < 0.01) between WT and *ys* mutant determined by Duncan’s test.

It is worth mentioning that the *p*-coumaric acid acts as the common precursor for both flavonoid and lignin biosyntheses, and C3H and POD are two key enzymes in lignin biosynthetic pathway (Higuchi, 2006; van der Rest et al., 2006; Petrussa et al., 2013; Renault et al., 2017). Given the repression of two *SlC3Hs* and six *PODs* in yellow stigma of the *ys* mutant (**Table 2**), we speculated that the higher accumulation of *p*-coumaric acid in the *ys* mutant (**Figure 3B**) may result from the reduced lignin biosynthesis. To test this hypothesis, we compared the levels of caffeic acid, the downstream production of *p*-coumaric acid catalyzed by C3H and also a biosynthetic precursor of lignin (**Figure 6**) (Higuchi, 2006; Renault et al., 2017), and lignin in the stigmas at anthesis stage between WT and *ys* mutant. As expected, the caffeic acid and lignin contents were 7.6- and 8.5-fold decreased in the *ys* mutant compared to those of WT, respectively (**Figure 7** and Supplementary Table S3), suggesting that the reduced expressions of two *SlC3Hs* and six *PODs* might inhibit the biosynthesis of lignin, leading to a change in the direction of the metabolic flow of *p*-coumaric acid from lignin to flavonoid biosynthetic pathway, which eventually resulted in a higher naringenin chalcone level (**Figure 3B**). These observations indicated that the two *SlC3Hs* and six *PODs* may function as negative regulators in naringenin chalcone biosynthesis, which could play a positive role in yellow stigma formation in the *ys* mutant.

## Discussion

The worldwide popularity of tomato as a fruit vegetable is due to its unique taste and high nutritional value, which are principally contributed by various pigments and secondary metabolites, such as flavonoids. Tomato fruits that display diverse colors upon maturation, are developed from floral organ, however, the colors of the different parts of flower remain essentially unchanged. For example, sepal, ovary, style, and stigma are green, while petal and anther are yellow all the time. Flower color plays a crucial role in attracting specific pollinators to facilitate pollination, which is essential for fertilization and subsequent fruit development. Therefore, it is important to better understand the mechanisms of pigmentation in different floral parts. However, previous studies with respect to regulatory mechanism of pigmentation in tomato have mainly been focused on fruits, and rarely on flowers or stigmas. In this study, we identified a *ys* mutant with unusual “yellow stigma” phenotype through EMS mutagenesis (**Figure 2**) (Saito et al., 2011). This study provides a useful basis for understanding the mechanism of pigment regulation in tomato stigma.

Chlorophylls, carotenoids, and flavonoids are the main three kinds of pigments in tomato (Ono et al., 2006; Tanaka and Brugliera, 2013). The color of ripe tomato fruit is mainly determined by carotenoids and flavonoids, with the exception of specific genotypes (Ballester et al., 2010). While in this study, we found that chlorophylls and flavonoids are predominantly accumulated in the stigmas of both WT and *ys* mutant (Supplementary Table S3), suggesting that they play an important role in the formation of tomato stigma color. These observations also indicate an existence of similar as well as divergent pigment metabolism pathways between tomato stigma and fruit, while the flavonoids are involved in both processes. Furthermore, during the ripening process of tomato fruit, naringenin chalcone, one of the most abundant flavonoids in the fruit peel, is responsible for the yellow color at the breaker stage (Moco et al., 2006; Iijima et al., 2008; Ballester et al., 2010). In addition, the altered carotenoid compositions can also result in yellow color of fruit (Ballester et al., 2010). And in the stigma, naringenin chalcone functions as an activator for yellow stigma formation, owing to its significantly increased content detected in the *ys* mutant, but carotenoids do not affect stigma color because their accumulation is too much tiny and has no change between WT and *ys* (**Figure 3** and Supplementary Table S3), supporting that carotenoids play different roles in yellow color formations of tomato stigma and fruit, while flavonoid naringenin chalcone is key in these two processes.

Lignin, a major component of secondary cell wall, can provide mechanical strength and also defend the vascular plants against biotic and abiotic stresses (Senthil-Kumar et al., 2010; Bi et al., 2011; Liu et al., 2016; Wang et al., 2017). Lignin is formed via the phenylpropanoid pathway and share common biosynthetic precursors with flavonoids, such as phenylalanine, cinnamic acid, and *p*-coumaric acid (Higuchi, 2006; van der Rest et al., 2006; Petrussa et al., 2013; Renault et al., 2017). Reduced lignin biosynthesis can enhance the availability of these precursors and, thereby, stimulate the production of flavonoid compounds. For example, up-regulation of cinnamate 4-hydroxylase (C4H), an enzyme catalyzed the synthesis of *p*-coumaric acid from cinnamic acid, shows opposite effects on the levels of stem lignin and fruit flavonoid, especially naringenin and rutin (Millar et al., 2007). Down-regulation of cinnamoyl-CoA reductase (CCR), a key enzyme in the formation of lignin monomers, results in decreased lignin content and higher amounts of chlorogenic acid, rutin and kaempferol rutinoside in tomato (van der Rest et al., 2006). And in this article, through RNA-Seq and biochemical analyses, we demonstrated that repression of C3H and POD, which are thought to be essential for lignin biosynthesis (Quiroga et al., 2000; Franke et al., 2002; Higuchi, 2006), leads to reduced lignin accumulation and increased *p*-coumaric acid and naringenin chalcone levels (**Figures 3, 7** and **Table 2**), which has similar interaction model between lignin and flavonoid as previous studies, but different effects on flavonoid compounds.

In fact, the roles of *C3H* gene in flavonoid metabolism and plant color have been verified previously. For instance, *C3Hs* participate in the biosynthesis of chlorogenic acid (Mahesh et al., 2007; Kim Y.B. et al., 2013). And repression of *C3Hs* in *Arabidopsis* and maize increases the accumulation of anthocyanins, leading to purple coloration in their leaves (Abdulrazzak et al., 2006; Fornaléa et al., 2015). Moreover, extensive studies have been performed in the regulation of *POD* genes on plant responses to stress, but the involvement of *PODs* in plant color control has never been reported. Therefore, our results strengthen the theory of regulation of *C3Hs* in flavonoid pathway and provide a new perspective of the effect of *C3Hs* and *PODs* on naringenin chalcone, contributing to the yellowing of stigma color in tomato. As it stands now, our understanding of the biological functions of two *SlC3H1s* and six *PODs* is based on bioinformatic and biochemical analyses, therefore, further study is to be carried out to elucidate their specific roles in tomato stigma color formation by expression pattern analysis and genetic transformation study (RNAi and overexpression) in tomato. As such, our data provide valuable information for further functional characterization of yellow stigma phenotype.

Furthermore, the transcriptional regulation of flavonoid and lignin biosyntheses is controlled by some key TFs in plants. For instance, R2R3-MYB, bHLH, and WDR participate in flavonoid biosynthetic pathway (Xu et al., 2014, 2015), while KNOX, HD-ZIP, NAC, and R2R3-MYB are involved in the regulation of lignin (Du et al., 2011; Xu et al., 2012; Townsley et al., 2013; Liu et al., 2015). It appears that R2R3-MYB factors potentially play key roles in both pathways. Further, some members of this family have been recognized as positive regulators of flavonoid biosynthesis. For example, AtMYB11, AtMYB12, and AtMYB111 in *Arabidopsis* can activate flavonoid biosynthetic genes, and overexpression of *AtMYB11* or *AtMYB12* enhances the flavonol content (Mehrtens et al., 2005; Li et al., 2015; Liu et al., 2015). In tomato, deregulated expression of *SlMYB12* results in a decreased level of naringenin chalcone, leading to pink fruit color (Ballester et al., 2010). Moreover, several R2R3-MYB TFs have been identified as repressors of lignin biosynthetic pathway. For instance, ZmMYB31 and ZmMYB42 of maize suppress several lignin genes, thereby reducing the lignin content, and this effect redirects the phenylpropanoid metabolic flux toward the biosynthesis of flavonoid (Sonbol et al., 2009; Fornale et al., 2010). And MYBs from other species such as snapdragon (*Antirrhinum majus*), switchgrass (*Panicum virgatum*) and leucaena (*Leucaena leucocephala*) can also inhibit lignin biosynthesis (Liu et al., 2015). In our study, given the blocked lignin biosynthetic pathway and increased accumulation of flavonoids in the stigma of *ys* mutant (**Figures 3B, 7**), we speculate that the shift in the expression profile of *C3Hs* and *PODs* may also be regulated by R2R3-MYB TFs, however, this hypothesis needs to be confirmed in the future.

In addition, given the importance of stigma in pollination of flowering plants, we tried to explore the effect of this stigma color mutation on reproductive development in tomato, but disappointingly, we did not find any significant difference between WT and *ys* mutant in terms of stigma receptivity, pollen viability, pollination, and fruit set (data not shown). Even so, the discovery and description of *ys* phenotype have important scientific and theoretical significances in understanding the mechanism of tomato stigma color development, which has never been reported before our study, and improving the pigment regulation in tomato plants. Given that both flavonoids and lignin play important roles in plant responses to environmental stress (Senthil-Kumar et al., 2010; Petrussa et al., 2013; Zhang et al., 2015; Liu et al., 2016), and their contents are changed in the *ys* mutant as compared to those of WT (**Figures 3, 7**), in future work, we will explore the importance of stigma color in term of interaction between pigments and environmental factors in tomato.

## Author Contributions

YZ and YaL designed the experiments, YZ, GZ, JZ, and MS performed the experiments. YZ, GZ, YuL, and TM analyzed the data. YZ wrote the paper along with YaL. All authors reviewed the manuscript.

## Conflict of Interest Statement

The authors declare that the research was conducted in the absence of any commercial or financial relationships that could be construed as a potential conflict of interest.

## Abbreviations

ANS: anthocyanidin synthase
bHLH: basic helix-loop-helix
C3H: *p*-coumarate 3-hydroxylase
CHI: chalcone isomerase
CHS: chalcone synthase
DEG: differentially expressed gene
DFR: dihydroflavonol reductase
DGE: differentially gene expression
EMS: ethylmethane sulfonate
ER: endoplasmic reticulum
FDR: false discovery rate
F3′H: flavonoid 3′-hydroxylase
F3′5′H: flavonoid 3′,5′-hydroxylase
FLS: flavonol synthase
FPKM: fragments per kilobase of exon per million mapped fragments
FSII: flavone synthase II
GO: Gene Ontology
GT: glucosyltransferases
3GT: flavonoid-3-*O*-glucosyltransferase
5GT: flavonoid-5-glucosyltransferase
HPLC: high performance liquid chromatography
KEGG: Kyoto Encyclopedia of Genes and Genomes
LBGs: late biosynthetic genes in flavonoid biosynthetic pathway
LC-MS: liquid chromatograph-mass spectrometer
OGD: 2-oxoglutarate-dependent dioxygenases
PDA: photo-diode array
POD: peroxidase
qRT-PCR: quantitative real-time PCR
RT: flavonoid 3-*O*-glucoside-rhamnosyltransferase
WDR: WD40-repeat
WT: wild-type
*ys*: *yellow stigma*
