# Supplementary material for: Transcriptome Profiling of Tomato Uncovers an Involvement of Cytochrome P450s and Peroxidases in Stigma Color Formation
Source: Front Plant Sci. 2017 May 31;8:897. doi: 10.3389/fpls.2017.00897 (PMC5449478; doi:10.3389/fpls.2017.00897)
Supplement: Supplementary file 5 [file Data_Sheet_1.PDF]

Title: Transcriptome profiling of tomato uncovers an involvement of cytochrome P450s and peroxidases in stigma color formation

Yan Zhang[1,2], Guiye Zhao[1,2], Yushun Li[1,2], Jie Zhang[1,2], Meijing Shi[1,2], Tayeb Muhammad[1,2], Yan Liang[1,2 *]

Supplementary Materials

Figure S1. The quality of the raw reads.

Figure S2. KEGG graph of flavonoid biosynthesis pathway.

Table S1. Identification of flavonoid compounds and polyphenols in LC-MS analysis.

Table S2. List of primers used for qRT-PCR verification.

Table S3. Chlorophyll, carotenoid, flavonoid and polyphenol contents in stigmas at the anthesis stage of WT and *ys* mutant.

Table S4. Summary of the transcriptome assembly.

Table S5. The whole normalized transcriptome data.

Table S6. List of genes that were differentially expressed in stigmas between *ys* mutant and WT.

Table S7. List of DEGs classified into GO terms.

Table S8. List of DEGs mapped to KEGG pathways.

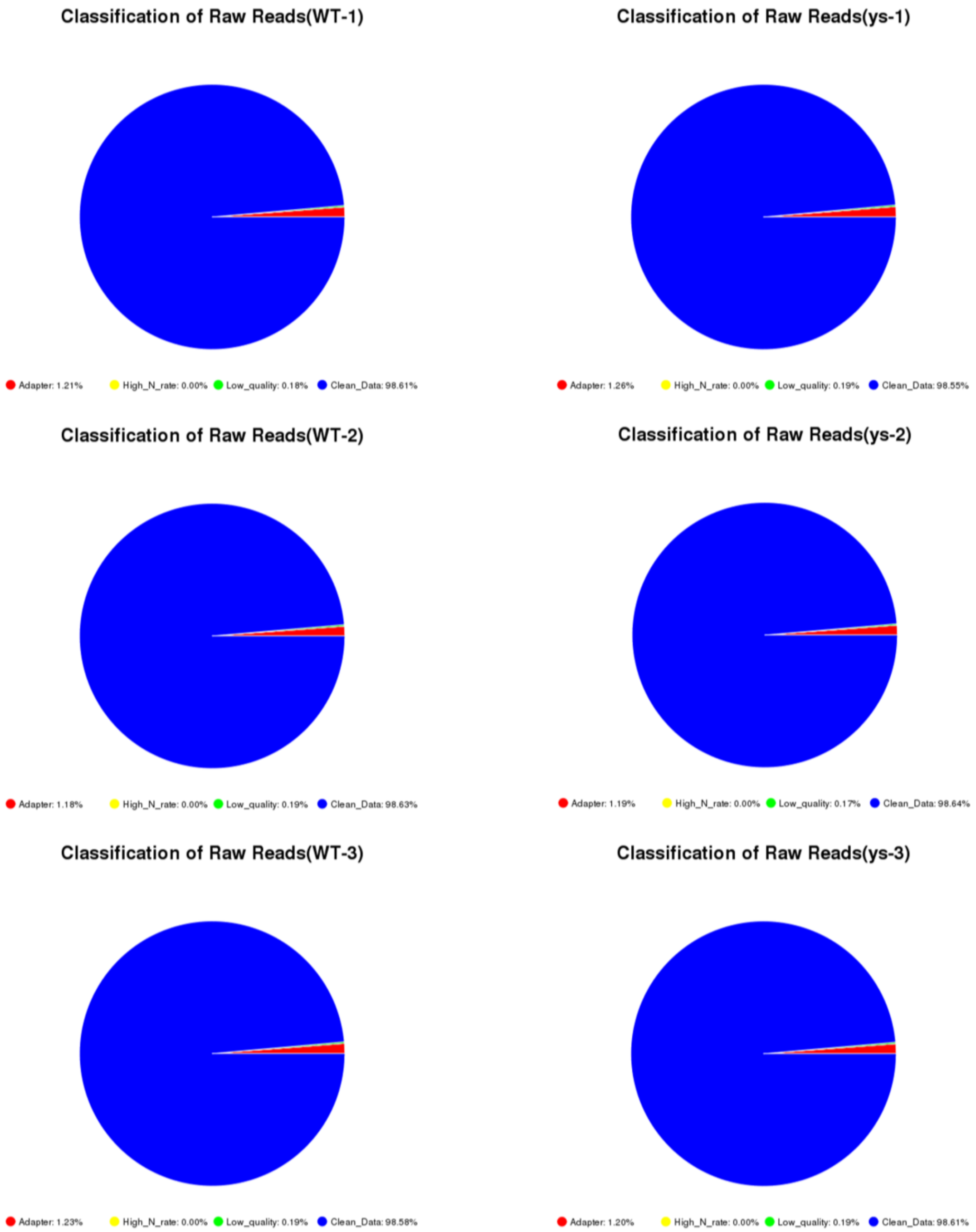

Figure S1. The quality of the raw reads.

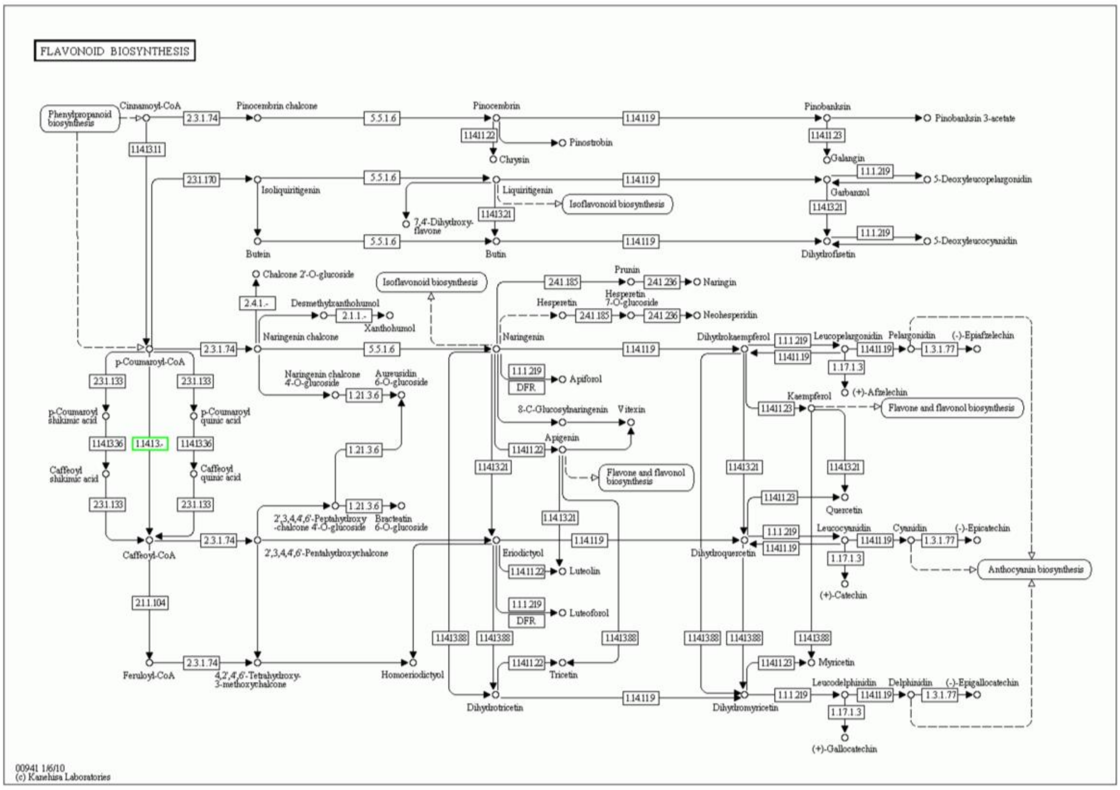

Figure S2. KEGG graph of flavonoid biosynthesis pathway.

Down-regulated and non-change genes are shown in green and black boxes, respectively. ‘‘1.14.13.-’’ in the green box indicates the two cytochrome P450 genes *SlC3H1* and *SlC3H2*.

Table S1. Identification of flavonoid compounds and polyphenols in LC-MS analysis.

**Flavonoid or polyphenol**	**Retention time (min)**	**$(M + H)^+$ (*m/z*)**
p-Coumaric acid	22.7	165
Caffeic acid	13.2	181
Naringenin chalcone	42.1	273
Kaempferol-3-rutinoside	31.5	595
Quercetin-3-rutinoside (Rutin)	26.8	611

Table S2. List of primers used for qRT-PCR verification.

Primer	Sequence
Solyc12g008900-F	GGTAAAAGGATTGAAGTATGTAGG
Solyc12g008900-R	TCTTCTTCTGGTATTATTGCTGA
Solyc03g093890-F	AACCACAAGTTTTTTTTCTTTCTGA
Solyc03g093890-R	TCCATCTTAGTCTACAACTTTTCCC
Solyc08g076820-F	GATGATTGTGACGGTGGAAAGAGA
Solyc08g076820-R	TAAAGTGGTGACATTGAGGTGAAGG
Solyc08g074620-F	GACAACACAAGAGAAAAATGAGCAA
Solyc08g074620-R	CCTCCAACAGTTCAGTTATCGCC
Solyc10g055760-F	ACACAGCTCTCCTTGTTCAACAAC
Solyc10g055760-R	TAACCTATCATCATCTACTTCATCCT
Solyc06g064840-F	AACTGCTGAAACCTCTAATGCTTGC
Solyc06g064840-R	ACTGTAATCTTGTCCTCCTGCTGCT
Solyc10g078220-F	GTGAATAATGCGGAACTTGCTAAA
Solyc10g078220-R	CGTTTTACTATCATCACCAGGCTT
Solyc10g078230-F	TTGCTAGAGAAAAATCTGGAGAA
Solyc10g078230-R	ATTAGTGGAGTTGGAGGGTGC
Solyc07g056510-F	ACCCTATCCATAAGCAAATCCCA
Solyc07g056510-R	CTCCTCCAAAGAAAGTCTTGTCAC
Solyc12g099140-F	ATTTGCCAGGAAGAACCGATAAC
Solyc12g099140-R	ATTCTCTCAACTAGCCTTGGTATCC
Solyc02g021680-F	AACAAGTGGAAAGGAGCAGGACAG
Solyc02g021680-R	TCTTTAGAAAACTCCTCGTCATCGC
Solyc01g109120-F	TAATAGTATGGGAGCGATGTTGC
Solyc01g109120-R	GATTTTACATAAGGCGAACCAGA
Solyc07g056670-F	TCCATTGATTGACCTCTCTAAACCC
Solyc07g056670-R	TGTTTATTGCCATAGCCAAAAGGAT
Solyc06g083170-F	CAAAAACTCAAGCCTAATGATGTGT
Solyc06g083170-R	TTCCATTGTTGCTCCCATTACCTT
Solyc01g100460-F	ATCTCTGCCATTTCCATCACTTT
Solyc01g100460-R	TCTCATCCATTCCTGCATACCTT
Solyc04g077010-F	TCGTCATCAAATAGCACTCGGAA
Solyc04g077010-R	CATAGGTTCCTGCGATGACTGTG
EF-1α-F	GACAGGCGTTCAGGTAAGG
EF-1α-R	CCAATGGAGGGTATTCAGC

Table S3. Chlorophyll, carotenoid, flavonoid and polyphenol contents in stigmas at the anthesis stage of WT and *ys* mutant.

	WT	*ys*
Chlorophylls (mg/kg FW)	141.29±6.17	151.17±12.37
Carotenoids (mg/kg FW)	3.89±0.18	3.39±0.35
p-Coumaric acid (mg/kg FW)	0.73±0.09	3.14±0.25
Caffeic acid (mg/kg FW)	7.56±1.08	1.08±0.32
Naringenin chalcone (mg/kg FW)	26.96±4.36	241.35±32.90
Kaempferol-3-rutinoside (mg/kg FW)	3.97±0.37	3.54±0.35
Quercetin-3-rutinoside (Rutin) (mg/kg FW)	8.68±0.38	9.37±0.76

Table S4. Summary of the transcriptome assembly.

**Samples**	**WT_rep1**	**WT_rep2**	**WT_rep3**	***ys*_rep1**	***ys*_rep2**	***ys*_rep3**
**Raw reads**	42,345,844	43,325,758	45,219,778	50,270,562	50,517,394	50,797,554
**Clean reads (%)**	41,758,186 (98.61%)	42,731,628 (98.63%)	44,582,128 (98.59%)	49,540,710 (98.55%)	49,827,948 (98.64%)	50,091,488 (98.61%)
**Total nucleotides (nt)**	5,219,773,250	5,341,453,500	5,572,766,000	6,192,588,750	6,228,493,500	6,261,436,000
**Q20 (%)**	94.62%	94.40%	94.52%	94.46%	94.79%	94.47%
**GC(%)**	42.99%	42.87%	42.85%	42.76%	42.86%	42.75%
**Mapped clean reads (%)**	38,311,300 (91.75%)	39,619,847 (92.72%)	41,150,451 (92.30%)	45,926,719 (92.71%)	46,430,696 (93.18%)	46,568,353 (92.97%)
**Unique mapped clean reads (%)**	38,080,524 (91.19%)	39,456,387 (92.34%)	40,977,295 (91.91%)	45,697,457 (92.24%)	46,268,204 (92.86%)	46,401,647 (92.63%)
